# Early developmental changes in the timing of turn-taking: a longitudinal study of mother–infant interaction

**DOI:** 10.3389/fpsyg.2015.01492

**Published:** 2015-09-29

**Authors:** Elma E. Hilbrink, Merideth Gattis, Stephen C. Levinson

**Affiliations:** ^1^Language and Cognition Department, Max Planck Institute for PsycholinguisticsNijmegen, Netherlands; ^2^School of Psychology, Cardiff UniversityCardiff, UK

**Keywords:** turn-taking, mother–infant interaction, infants, timing, communicative development

## Abstract

To accomplish a smooth transition in conversation from one speaker to the next, a tight coordination of interaction between speakers is required. Recent studies of adult conversation suggest that this close timing of interaction may well be a universal feature of conversation. In the present paper, we set out to assess the development of this close timing of turns in infancy in vocal exchanges between mothers and infants. Previous research has demonstrated an early sensitivity to timing in interactions (e.g., Murray and Trevarthen, 1985). In contrast, less is known about infants’ abilities to produce turns in a timely manner and existing findings are rather patchy. We conducted a longitudinal study of 12 mother–infant dyads in free-play interactions at the ages of 3, 4, 5, 9, 12, and 18 months. Based on existing work and the predictions made by the Interaction Engine Hypothesis (Levinson, 2006), we expected that infants would begin to develop the temporal properties of turn-taking early in infancy but that their timing of turns would slow down at 12 months, which is around the time when infants start to produce their first words. Findings were consistent with our predictions: infants were relatively fast at timing their turn early in infancy but slowed down toward the end of the first year. Furthermore, the changes observed in infants’ turn-timing skills were not caused by changes in maternal timing, which remained stable across the 3–18 months period. However, the slowing down of turn-timing started somewhat earlier than predicted: at 9 months.

## Introduction

For a conversation to run smoothly, a tight coordination of interaction between speakers is required. In their seminal paper on the organization of turn-taking in conversation Sacks et al. (1974) noted that in conversation mostly one speaker talks at a time, that occurrences of overlap (i.e., more than one speaker at the time) are common, but brief, and that the vast majority of turn transitions (i.e., the switch from one speaker to the next) are characterized by either no gap and no overlap or by a slight gap or slight overlap. Moreover, a recent study comparing across a variety of languages demonstrated that this close-timing of turns might be universal (Stivers et al., 2009). Stivers et al. (2009) compared the turn transitions in naturalistic conversation across 10 diverse languages that differed, amongst other things, in word order, sound structure, and grammatical options. They found that, despite of some variation in the overall distributions, turn transitions in all of the languages had a mode between 0 and 200 ms. This is remarkably fast considering that it takes at least 600 ms to plan a short turn at talk (Levinson, 2013). This suggests that in order to produce smooth transitions from one speaker to the next, speakers need to simultaneously listen to the other speaker, plan their own turn, and predict when to launch that turn. It therefore comes as no surprise that children who are still in the process of acquiring language are much slower than adults. Garvey and Berninger (1981), for example, reported a mean gap duration of 1100–1800 ms in preschoolers engaged in child–child conversation. Casillas et al. (under review) reported a median gap duration in mother child question–answer pairs of 587 ms for children between the ages of 2;8 and 3;5.

The Interaction Engine hypothesis suggests that the infrastructure for this remarkably tight coordination underlying conversation is based on our sensitivity to the timing of turns and our ability to anticipate and recognize others’ communicative intentions (Levinson, 2006). Moreover, this social interactional infrastructure is thought to be present early in infancy, before infants have acquired language, and is hypothesized to be the foundation for communicative turn-taking. Thus, even though preschool-aged children have been shown to be slow compared to adults, the Interaction Engine hypothesis suggests that preverbal infants already have (parts of) the necessary interactional infrastructure at their disposal. More specifically, the Interaction Engine hypothesis suggests that the temporal aspect of this infrastructure, i.e., turn-timing, develops early in infancy. Like scholars such as, e.g., Bruner (1975, 1981) and Bateson (1975) this hypothesis views early mother–infant exchanges as proto-conversations and places great emphasis on the role of early interactional skill. But, while Bruner focuses mostly on speech acts, or understanding of communicative intentions, the Interaction Engine hypothesis ascribes important roles to both the understanding of communicative intentions as well as the temporal aspect of these early exchanges. The present paper aims to explore the development of the temporal aspect of turn-taking, i.e., turn-timing, during infancy in naturalistic interactions.

Infants spend a substantial portion of their awake-time in face-to-face interactions with their caregivers and it is this face-to-face conversational setting that provides an important context for infants to start acquiring language. Moreover, well-before infants have acquired language they start to interact in social exchanges characterized by turn-taking patterns, such as peek-a-boo games and give and take sequences (e.g., Bates et al., 1975; Ratner and Bruner, 1978; Rochat et al., 1999). Thus, it is in these face-to-face interactions where one might expect to observe infants’ earliest communicative abilities. Indeed, Kaye suggested for example that the burst-pause patterns observed in sucking during feeding and in facial expressions in mother–infant face-to-face interactions, resemble the turn-taking patterns in communication and could be the basis for acquiring communicative turn-taking abilities (Kaye, 1977; Kaye and Fogel, 1980). Furthermore, a recent study on face-to-face interaction in the first 6 months of life showed that mother and infant spend most of their time in unilateral communication, i.e., where mother tries to engage the infant but the infant is not attending, however, with increasing infant age the time they spend in symmetrical communication, i.e., being mutually engaged with a joint focus of attention, increases (Hsu and Fogel, 2003). These mutually engaged interactions can consist of behaviors in multiple modalities such as vocalizations, gaze and smiles, which have been shown to be temporally ordered. For example, Hsu et al. (2001) found that infants’ speech-like sounds occurred more often when their mothers were smiling, when infants were looking at their mothers, and also when they themselves were smiling. Moreover, speech-like vocalizations, compared to non-speech-like vocalizations, were more likely to be preceded by maternal smiling, indicating a temporal ordering of these interactional behaviors.

While the above studies demonstrate the existence of a general temporal coordination, they do not allow for the fine-grained temporal analyses that are common in adult studies on timing of turns in conversation (e.g., ten Bosch et al., 2005; Stivers et al., 2009). In order to assess this precise timing of turns in mother–infant interactions in the present study we specifically focused on vocal exchanges. Various studies on vocal exchanges in naturalistic interactions suggest that these early vocal exchanges between mothers and infants can be described as conversation-like (Bateson, 1975; Snow, 1977). Furthermore, several experimental studies, in which it was manipulated whether infants received contingent responses (i.e., responses related to the infants’ behavior) or yoked (non-contingent) responses, have demonstrated that contingent responding has positive effects on the quality of infant vocalizations and facilitates turn-taking behavior in vocal exchanges between mothers and infants (e.g., Bloom et al., 1987; Bloom, 1988; Masataka, 1993; Goldstein et al., 2003). However, these studies are solely based on experimental manipulation and only assess short-term effects. Nevertheless, various scholars have suggested an important role for early turn-taking behaviors in the development of language. For example Ginsburg and Kilbourne (1988) state: “*The possibility has become widely accepted over the past decade that the temporal patterning of social interchanges involving the young infant has important implications for linguistic development”* (p. 221). Thus infants’ (vocal) turn-taking behaviors in face-to-face interactions are suggested to be among the earliest communicative abilities that infants might demonstrate.

Some experimental evidence indicates that infants are sensitive to the timing of turn-taking in social exchanges. Striano et al. (2006) adapted a setup originally designed by Murray and Trevarthen (1985), in which mother and infant interact via screens, by adding a device to the video setup that allowed the ongoing interaction to be delayed by 1 s. In the original setup infants saw a live interaction and a replay of the interaction, while in the adapted version the interaction was delayed (online) by only 1 s. Similar to Murray and Trevarthen (1985), Striano et al. (2006) found that 3- and 6- month-olds gazed less at their mothers during the delayed interaction compared to the live interaction. In addition, an eye tracking study by Thorgrimsson (2014) has shown that when 1-year-olds observe two people in a face-to-face context, they are quicker to shift their gaze to person (B) when person (A) uttered a sentence compared to when person (A) emitted a non-speech sound (e.g., throat clearing, singing). In both conditions person (B) never responded, thus infants could not have learned what to expect during the experiment. This indicates that 1-year-olds expect speech to provoke a response. Several eye-tracking studies by Casillas and Frank (2013) have taken this paradigm one step further and have demonstrated that 1-year-olds are able to anticipate the upcoming turn when observing two adults or two puppets having a conversation. Together, these studies demonstrate that from early on in the first year, before infants have acquired language, infants are sensitive to changes in the timing of social interactions.

In contrast to infants’ perception and comprehension of timing, less is known about infants’ abilities to produce turns in a timely manner, and existing findings are rather patchy. A few studies have tried to assess turn-timing structure in infancy. Bateson (1975), for example, provided a detailed description of five interactions between one mother and her baby recorded between the ages of 1.5 and 3.5 months. Her analyses suggested that mother and infant alternated turns and that this alternation of turns seemed mutual. Both mother and infant left longer silences between two consecutive utterances made by themselves compared to when they responded to each other’s utterances; however, this pattern only reached significance for the mother. Naturalistic studies further suggest that infants start out producing a large portion of their vocalizations in overlap with their mothers’ utterances. The amount of overlapping vocalizations is said to decrease in favor of a more alternation-like pattern from around 4 months of age (Elias et al., 1986; Ginsburg and Kilbourne, 1988; Elias and Broerse, 1996). However, a study by Rutter and Durkin (1987) assessing turn-timing from 9 to 36 months reported an increase in overlapping vocalizations from 9 to 24 months. In addition, studies assessing precise timing have also resulted in mixed findings. **Table 1** summarizes several of these earlier studies and their methods. The mean gap durations reported between the ages of 1 and 4 months range from 800 to 1370 ms (Bateson, 1975; Elias et al., 1986; Beebe et al., 1988). Jasnow and Feldstein (1986) reported a gap duration for 9-month-olds of 875 ms. Whether one would conclude from this that infants remain stable in their timing or start to speed up at 9 months depends on which of the studies you rely on for the gap durations at 1–4 months. To complicate things further, studies have differed on how to record the timing. Bateson (1975) for example reported maternal and infant gap durations which were recorded from the onset of the other’s utterance to the onset of their own utterance, i.e., these included not just the transition from one speaker to the next but also the duration of the previous utterance. Others chose to look at the actual silence between two utterances. In other words they measured the time between the end of the utterance of one speaker to the beginning of the utterance of the next speaker (Elias et al., 1986; Jasnow and Feldstein, 1986; Beebe et al., 1988). Together, these findings demonstrate that the developmental picture of infant turn-timing is far from clear. Furthermore, previous findings are mostly based on only one age group or on cross-sectional samples. Longitudinal designs tracking development over extended periods of time could provide valuable insights about stability and developmental change in turn-timing skills. A few longitudinal studies exist, but these studies are based on small samples including 1–3 children and/or cover a short period of time, i.e., 3–5 months (e.g., Bateson, 1975; Snow, 1977; Ginsburg and Kilbourne, 1988).

**Table 1:** Summary of several studies assessing precise turn-timing in infancy.

Study	Bateson, 1975	Elias et al., 1986	Jasnow and Feldstein, 1986	Beebe et al., 1988
Age (in months)	1.5–3.5	3–4	9	4
*N*	1	6	29	15
Average gap duration in ms (infant)	1370^∗^	1200	875	800
Average gap duration in ms (mother)	1430^∗^	750	775	700

*^∗^Includes preceding utterance duration*.

In addition to the questions about the developmental trajectory, considerable debate exists with respect to whether these vocal exchanges between mothers and their infants are reciprocally structured. Or whether in fact infants are randomly vocalizing while mothers are responsible for establishing a turn-taking structure, with possible observed changes due to changes in maternal behavior. Snow (1977) suggested that mothers are mainly responsible for maintaining the conversational structure. Anderson et al. (1977) found evidence for reciprocity in vocal exchanges at 3 months of age, while Rosenthal (1982) observed reciprocity in vocal interactions of neonates and their mothers. Furthermore, Jasnow and Feldstein (1986), Beebe et al. (1988), Jaffe et al. (2001) conducted a series of studies to assess mothers’ and infants’ capacity for interpersonal accommodation to gap durations (i.e., whether the moment of silence between speaker transitions is sensitive to the partner’s timing). The findings of these studies suggest that by 9 months of age infants’ gap durations were influenced by their mothers’ gap durations and vice versa. Jasnow and Feldstein (1986) called this interpersonal accommodation. Furthermore, they found that 4-month-olds change the length of their gap durations depending on who they interact with. For example, 4-month-olds left longer pauses when interacting with their mothers compared to when they were interacting with a stranger. This finding suggests that, contrary to Snow (1977), even 4-month-olds might be accommodating their gap durations.

There remains then considerable uncertainty about the development of turn-timing in preverbal infants. Especially with regards to their abilities to produce turns in a timely fashion and to reciprocally structure vocal exchanges. Existing findings on mother–infant vocal turn-timing are fragmented and mostly cross-sectional. Research, and especially longitudinal research, that tracks the development of turn-timing from early in infancy until the ages at which infants first start to produce language is still lacking. Moreover, studies have focused on assessing either overlapping vocalizations or precise gap durations but not both. The existing findings on the amount of overlapping vocalizations suggests a possible early decrease of overlapping vocalizations, while the various reports on infants’ gap duration suggests either stability of gap durations across age or a decrease in gap durations. This highlights the possibility that the developmental pattern for the amount of overlap might be different from the developmental pattern for gap durations. Therefore, to obtain a complete developmental picture research is needed that looks at both overlap and gaps. In studies of adult turn-timing overlap and gap measurements are often combined in a single floor-transfer offset measure where overlaps are treated as negative gaps, on the presumption that adult speakers are aiming at close transition times and may inadvertently come in early. However, in studies of infant turn-timing we cannot make the same assumption. Thus, in addition to the possibility that overlap and gap might show different developmental patterns, we can also not assume that infants, like adults, aim at close-transition times. Therefore, the present study set out to explore the development of turn-timing in a longitudinal study of mother–infant interaction between the ages of 3- and 18- months, by assessing, in contrast to earlier developmental studies, both gaps and overlaps. But, contrary to studies on adult turn-timing, we analyzed the overlap and gap durations as separate measurements.

**FIGURE 1:**
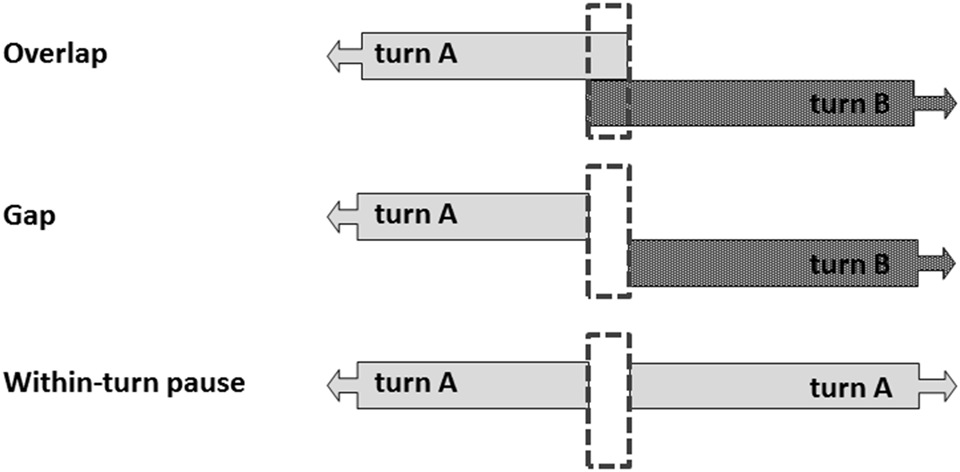
**A turn transition can consist of an overlap, a gap or if no turn transition occurs, a within-turn pause can occur**.

Specifically, the present study aimed to describe the developmental pattern of infants’ productive turn-timing abilities, including overlaps, gaps and within-turn pauses, i.e., the silence between two consecutive parts of the same turn of one interlocutor (see **Figure 1** for definitions). Furthermore we aimed to assess whether the observed mother–infant turn-timing patterns were reciprocally structured. We therefore conducted a longitudinal study of 12 mother–infant dyads in free-play interactions at six ages between 3 and 18 months. Based on previous work we expected that infants would begin to develop the temporal properties of turn-taking early in infancy. However, based on earlier work with older children and due to the complex nature of achieving smooth turn transitions we expected that at 12 months, i.e., around the time of the onset of language production, infant turn-timing would slow down (Garvey and Berninger, 1981; Casillas et al., under review). These predictions are consistent with the predictions of the Interaction Engine Hypothesis. This hypothesis suggests that preverbal infants acquire the temporal properties of conversational turn-taking early in infancy and that once infants start using language their turn-timing will slow down due to the need to integrate their developing linguistic skills with the existing interactional timing skills (Levinson, 2006). Furthermore, based on earlier work by Anderson et al. (1977), Jasnow and Feldstein (1986), Beebe et al. (1988), Jaffe et al. (2001) we expected that the observed turn-timing patterns were reciprocally structured at all ages.

## Materials and Methods

### Participants

For the present study free-play recordings from 12 mother–infant dyads (seven female) were analyzed when the infants were 3, 4, 5, 9, 12, and 18 months. Infants were originally recruited as part of the First Steps longitudinal study (Ellis-Davies et al., 2012). First Steps followed 39 healthy infants (18 female) from birth to 18 months. The study consisted of monthly testing on a variety of measures, free-play observations, diary data and questionnaires from 2 months onward. Infants were born to full gestation. All procedures for data collection were reviewed and approved by the Southeast Wales Research Ethics Committee of NHS Wales. In addition, all procedures and data analyses used in the present study were approved by the Radboud University Ethics committee under the research program: *INTERACT-Developmental studies* (Hilbrink and Levinson; project code ECG2012-2711-065). Written consent was obtained from all parents before the start of the study. Parents’ level of education ranged from secondary school to postgraduate. Demographics on the full sample are available in Ellis-Davies et al. (2012). The level of education and maternal age of the 12 mothers in the present sample are comparable to the level of education and maternal age of the full sample (see **Table 2**). Parents were recruited during pregnancy through newspaper and web announcements and local events for expecting parents. The ages analyzed in the present study were chosen based on previous literature indicating an important transition around 4 months of age from vocalizing in overlap to a more turn-taking like pattern (Ginsburg and Kilbourne, 1988) and on studies suggesting important changes in communicative development emerging at 9 months (Bakeman and Adamson, 1984; Carpenter et al., 1998). In addition, ages were also chosen based on the predictions made by the Interaction Engine Hypothesis that the temporal properties of turn-taking are realized early in infancy and are expected to change once infants start to acquire language (Levinson, 2006).

**Table 2:** Demographic characteristics of the total sample and the current sample.

Maternal characteristics	Total sample (%)	Total sample *N*	Current sample (%)	Current sample *N*
Age in years at recruitment into the study				
16–20	0	0	0	0
21–25	11.11	4	8	1
26–30	25.00	9	25	3
31–35	33.33	12	33	4
36–40	30.56	11	33	4
Highest level of education attained				
High school	22.22	8	33	4
Undergraduate degree	52.78	19	50	6
Postgraduate degree	25.00	9	17	2

### Procedure and Design

Mothers brought their infants to a ‘mum and baby breakfast’ at a community center or the university. For the present study only the recordings of the 10-min free-play mother–infant interactions were used. After mother and infant were seated mothers were asked to play with their infants as they would do at home. Although toys were available, mothers were not specifically instructed to use them. The experimenter would then leave the room and return after 10 min had passed.

### Apparatus

The free-play interactions took place in a quiet room inside a tent, at 3, 4, and 5 months, or in a playpen, at 9, 12, and 18 months. Both setups were adapted to the needs of the infants at the various ages. To create privacy and consistency of visual environment, the mother–infant interactions at the younger ages (i.e., 3, 4, and 5 months) were conducted in a colorful tent. Infants were seated in a baby seat and the mother sat facing the infant. Three baskets containing age-appropriate toys, including soft toys, books and rattles were provided. The interactions were recorded using two static cameras, one filming the mother and the other filming the infant. Two microphones (AKG C1000S) recorded the sound from the same location as the two static cameras. A third camera was mounted on the mother’s head with a headband allowing us to capture where the mother was looking. The signals of each of the three cameras were combined using a quad splitter, which resulted in a single time-synced split-screen video record.

At the older ages (i.e., 9, 12, and 18 months) the set-up was similar but because infants were able to sit upright and to move somewhat more, the tent was replaced by a playpen. This setup allowed infants to sit upright in a supportive seat within reach of the three baskets with toys. Interactions in the playpen were recorded using four static cameras: one capturing the infant, one capturing the mother, one capturing both mother and infant, and one capturing a bird’s eye view of the playpen. The signals from each of these cameras were combined by a quad splitter, which resulted in a single time-synced split-screen video.

### Transcription

The mother–infant interaction recordings of 12 mother–infant dyads were transcribed at six time points: 3, 4, 5, 9, 12, and 18 months of age using ELAN video annotation software (Sloetjes and Wittenburg, 2008). The 10-min recordings were transcribed for all maternal speech and for all infant sounds. Grunting, distress sounds and involuntary sounds, such as hiccups and sneezes, were excluded from analyses (e.g., Hsu et al., 2001). Maternal responses to involuntary infant sounds were, however, included as mothers often treated these sounds as communicative.

To calculate interrater reliability two recordings at each age were transcribed by another transcriber. With regards to the number of vocalizations identified for the infants the intraclass correlation (ICC) was 0.81. The percentage of agreement of a vocalization being a vocalization and not for example a distress sound was 76%. With regards to the timing of a vocalization, coders had to agree within a time window of two frames, i.e., 80 ms. The percentage of agreement for the time at which a vocalization started or ended was calculated based on all the vocalizations the coders agreed on being a vocalization. The agreement between coders for the time at which a vocalization started was 92% and the percentage of agreement for when a vocalization ended was 82%. For the number of utterances made by the mothers the ICC was 0.82. The percentage of agreement of an utterance being an utterance was 95%. The percentage of agreement for when an utterance started was 91% and for when an utterance ended was 86%.

## Results

All turn transitions, both transitions from mother to infant and from infant to mother, were extracted from the transcriptions. A turn transition was defined as any switch from a maternal utterance to an infant vocalization or vice versa (see **Figure 1**). This resulted in 8555 turn transitions. As can be seen in **Table 3**, some individual variation exists with regards to the number of turn transitions across dyads at each age. An infant gap was defined as the gap between a maternal utterance and a vocalization from the infant, i.e., the onset of an infant vocalization minus the offset of the preceding maternal utterance. Maternal gap was measured in a similar way: the onset of the maternal utterance minus the offset of the preceding infant vocalization. Infant overlap was defined as transitions in which the infant started to vocalize when the mother had not yet finished speaking and maternal overlap was defined as whenever the mother started speaking when the infant had not yet finished vocalizing. Infant overlap was measured in the same way as infant gap durations but resulted, because of the overlap, in negative durations. Similarly, maternal overlap was measured in the same way as maternal gap duration. Furthermore, the moments of silence between two consecutive utterances or vocalizations by the same interlocutor, i.e., without the other interlocutor producing a turn in between, were also assessed. These within-turn pauses were measured in the same way as infant or maternal gap durations: the onset of an utterance or vocalization minus the offset of the preceding utterance or vocalization (see also **Figure 1** for the definitions).

**Table 3:** Number of turn transitions per dyad at each age.

	Age (in months)						Average across age
Dyad	3	4	5	9	12	18	
1	107	108	85	123	225	273	154
2	224	213	127	34	130	146	146
3	119	142	40	46	97	150	99
4	184	135	96	111	91	98	119
5	41	71	58	56	42	112	63
6	197	111	56	48	148	109	112
7	36	66	92	100	41	208	91
8	129	149	118	83	196	140	136
9	125	158	275	77	147	221	167
10	70	155	179	24	35	175	106
11	88	111	152	184	142	164	140
12	90	54	81	50	91	196	94
**Average across infants**	118	123	113	78	115	166	

To study the development of turn-timing in infancy we assessed three aspects of turn-timing. First we assessed the development of timing with regard to overlap. Next we assessed the development of infants’ ability to time turns with regard to gap durations. We analyzed these separately (unlike many studies of adult turn-timing) because the developmental trajectory of overlapping turn transitions might differ from the developmental trajectory of transitions containing gaps. The third and final aspect of turn-timing we assessed was the development of turn-timing as whole, (i.e., all turn transitions, overlaps, and gaps) to assess whether infants’ turn-timing differed from what would be expected if infants were vocalizing randomly in each age group.

In addition to studying the developmental trajectory of infants’ turn-timing skills, we also explored whether mother–infant turn-timing patterns were reciprocally structured or whether changes in maternal behavior could account for possible changes in infant turn-timing with age. Analyses were conducted in R Development Core Team (2012) using the LME4 package (Bates et al., 2012). For the linear mixed effect modeling we followed the same procedures used by Hoicka and Akhtar (2011) and Hilbrink et al. (2013). All effects are expressed as odds ratios; when the odds ratio of an event is greater than one, the event is more likely to happen than not. The dependent variables that were included in the models were duration of overlap, duration of gap, or duration of within-turn pause. The variables that were included in the models as fixed effects were infant age in months (3, 4, 5, 9, 12, 18) and whether the overlap or gap durations were produced by mother or infant, i.e., person. Infant ID was included as random effect.

### Overlap

**FIGURE 2:**
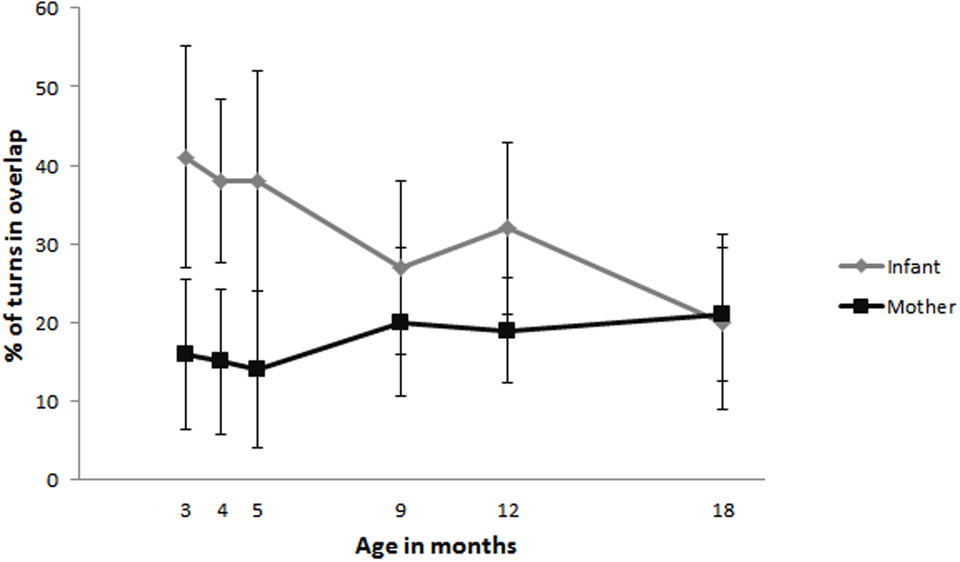
**Percentage of turns produced in overlap by infants (gray) and mothers (black)**.

**Figure 2** shows the percentage of overlap at each age. At 3, 4, and 5 months infants produce just over a third of their vocalizations in overlap with their mothers. Mothers, produce between 14 and 21% of their turns in overlap with their infants at these same ages. However, by the time infants are 18 months infants have decreased the amount of overlap to similar levels as their mothers: to roughly 20% of their turns.

**FIGURE 3:**
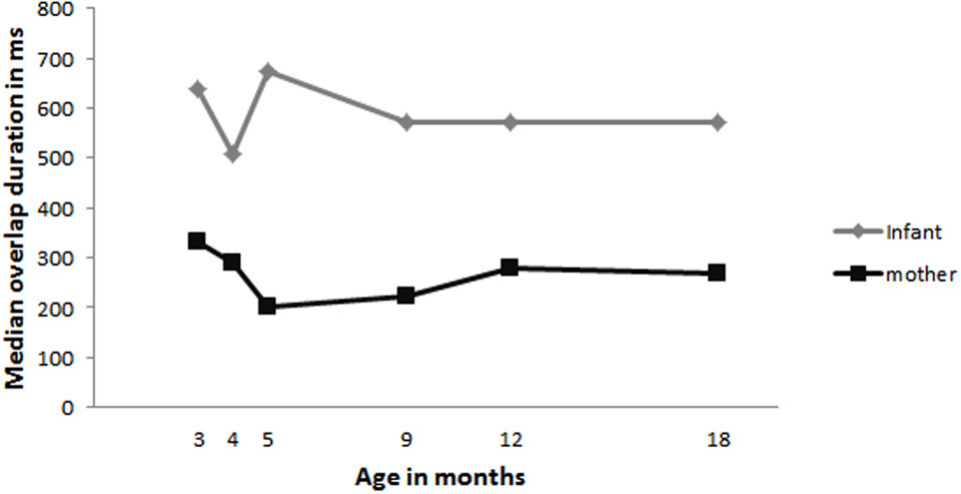
**Median durations of overlap for infant (gray) and mother (black)**. The closer the median is to zero the shorter the overlapping turn.

To assess whether infants also decreased the durations of their overlapping vocalizations we calculated the median durations of overlap (total number of overlapping turns: infant = 1180, mother = 840). Studies on adult turn-timing have demonstrated that brief overlaps occur often and that adults aim to launch their turn at the end of the previous turn which can result in brief overlap (Sacks et al., 1974; Jefferson, 2004). Thus if infants start decreasing the durations of overlapping turns this could indicate that they, like adults, are aiming for the end of the previous turn. **Figure 3** shows the infant and maternal median durations of overlap for each age. To assess whether infants and their mothers significantly change their duration of overlap we assessed all overlapping turn transitions using linear mixed modeling in R with duration of overlap as dependent variable and infant age and person (mother, infant) as fixed factors. We first build a base model with duration of overlap as dependent variable and infant ID as random effect. We compared this base model to models including age, person and an interaction of age and person. The base model was improved by adding age, χ^2^(1) = 18.97, *p* = 0.000, person, χ^2^(1) = 51.55, *p* = 0.000, and an interaction of age by person, χ^2^(1) = 10.27, *p* = 0.000. The final model included a significant interaction effect of age by person (model: *loglikehood* = -16402, *N* = 2020), *OR* = 1369895094, *p* = 0.001. To follow up this interaction we created separate models for mothers and infants. The separate analyses of data including only the overlap durations of the infants revealed no significant effect of age. The analyses on the data only including the maternal overlap durations did reveal a significant effect of age [χ^2^(1) = 13.93, *p* = 0.000; model: *loglikehood* = -6865.8]. Maternal overlap durations became significantly shorter with increasing infant age (*OR* = 366679967, *p* = 0.000).

### Gap

**FIGURE 4:**
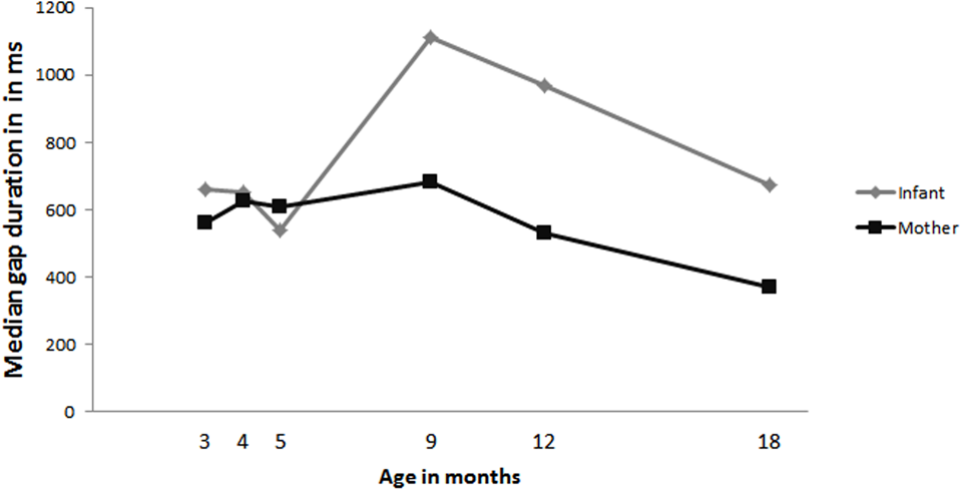
**Median gap durations for infant (gray) and mother (black)**.

To assess whether and how gap durations changed over time, infant and maternal median gap durations were calculated for each age (total number of gaps: infant = 2563, mother = 3992). See **Figure 4** for the median durations at each age and **Table 4** for the range of median gap durations observed at each age. To assess whether these gap durations changed significantly across age we used linear mixed modeling with duration of the gaps as dependent variable and age and person (mother, infant) as fixed factors. We first build a base model with duration of gap as dependent variable and infant ID as random effect. We compared this base model to models including age, person and an interaction of age and person. The base model significantly improved by adding person, χ^2^(1) = 27.34, *p* = 0.000, an interaction of person by age, χ^2^(2) = 1.23, *p* = 0.04 and a trend was found for age, χ^2^(1) = 3.26, *p* = 0.07. The final model (*loglikehood* = -58435, *N* = 6555) contained a significant effect of age (*OR* = 2987657, *p* = 0.01), namely, gap durations became significantly larger with increasing age, and a significant age by person interaction, *OR* = 1.246925e-08, *p* = 0.02).

**Table 4:** Range of median gap durations at each age in ms.

	Month 3	Month 4	Month 5	Month 9	Month 12	Month 18
Infant	345.5–902.5	326–921	323–1408	542.5–3297	615–1872	485–1270
Mother	372.5–905.5	412–938	391–1204	445–1005	210–817	135–1145.5

To follow up on the significant age by person interaction, we created separate models for mothers and infants. The separate analyses of data including only the gap durations for mother did not reveal a significant effect of age. The analyses on the data only including infant gap durations did reveal a significant effect of age [χ^2^(1) = 5.95, *p* = 0.01; model: *loglikehood* = -22928, *N* = 2563]. The effect of age indicates that infants’ gap durations increase with age (*OR* = 5025322, *p* = 0.01). Furthermore, inspection of the individual data of the infants showed, compared to the earlier ages, that all infants slowed down at some point between 9 and 12 months, with most of the infants being the slowest at 9 months (8 out of 12 infants). Based on the effect of age, the findings in **Figure 4** and our observations in the individual data we conducted exploratory follow-up analyses to assess the difference in infant gap durations between 5, 9, 12, and 18 months. To do this we ran the same linear mixed model analyses on infant gap durations but separately on data sets including only the data at 5 and 9 months of age, 5 and 12 months of age and 5 and 18 months of age. These analyses revealed an effect of age for the dataset including 5 and 9 months [χ^2^(1) = 25.18, *p* = 0.000; model: *loglikehood* = -5716.9, *N* = 630] and the dataset including 5 and 12 months [χ^2^(1) = 13.31, *p* = 0.000; model: *loglikehood* = -7001.3, *N* = 778]. No effect of age was found for the dataset including the infant gap durations at 5 and 18 months. Thus infant gap durations are significantly longer at 9 months compared to 5 months (*OR* = 2.806199e+96, *p* = 0.000) and at 12 months compared to 5 months (*OR* = 1.724077e+33, *p* = 0.000), but not at 18 months compared to 5 months.

### Reciprocity: Do Infants Equally Structure the Interaction?

Thus far the results indicate that infants get better in producing their turns with less overlap (i.e., they decrease in the amount of overlap produced) as they get older, and that infant gaps are relatively short at 3, 4, and 5 months, but increase with age. However, it is possible that the changes observed in infant timing are not due to infants’ changing turn-timing skills. Instead infants could be randomly producing turns, while mothers are trying to maintain a turn-taking structure by carefully timing their turns. For example mothers could change, as infants get older, in how long they wait for a vocalization from the infant. Especially when infants are older, mothers might expect a turn from their infant and might therefore increase their pauses between two consecutive utterances. Therefore we assessed, first, whether infants timed their turns significantly differently from what would be expected if they were vocalizing randomly at each age and, secondly, whether mothers changed their pause duration between two consecutive utterances.

**FIGURE 5:**
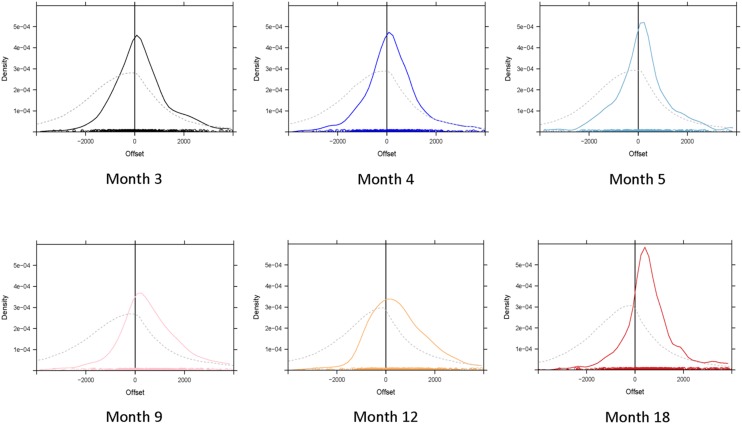
**Dashed lines are the random distributions, solid lines the actual distribution**.

To assess whether infants were randomly producing turns, we compared the observed distribution of infants’ turn transitions (i.e., including both gaps and overlaps) to random distributions. The random distributions of possible infant turn transitions were estimated by looking at points in the interaction when the infant could have taken a turn, but did not. Transitions were identified where utterance (A) came from the mother and then the next utterance (B) also came from the mother, without the infant taking a turn, i.e., maternal within-turn pauses. An infant producing turns at random points could have taken a turn within a window of time from the start of the mother’s turn to the end of the gap (any earlier or later would mean infants were transitioning from a different turn). Thus, the maternal turns were kept fixed, while the infants’ turn onsets varied randomly. The distribution was built by calculating the height of the distribution at a given distance from the end of the mother’s turn as the proportion of windows that included the given point. Because the time window, from the start of the mother’s turn until the end of the gap, always included the end of the maternal utterance, i.e., zero gap, the distribution has a peak around zero (see **Figure 5**). We then compared this ‘random infant’ distribution to the observed sample of infant turn transitions. This was done using a permutation test: i.e., the observations were randomly swapped between the observed sample and a random sample and then the difference in medians between these two new samples was calculated. A 1000 random infant samples were generated and permuted 10,000 times each with the observed sample. If the samples would have come from the same distribution, then the differences in medians should be normally distributed around zero. The probability of the given sample coming from the random distribution was calculated as the proportion of permutations resulting in a larger or equal difference in medians between the permuted samples than the actual difference, i.e., between the unpermuted samples. This probability was less than 1/10000000. In other words, out of 10 million permutations, none produced a difference in medians larger than the actual difference, suggesting that the actual distributions are significantly different (*p* < 0.00000001). Thus, at each age the infant turn transitions observed in our dataset differed significantly from the random distributions.

**FIGURE 6:**
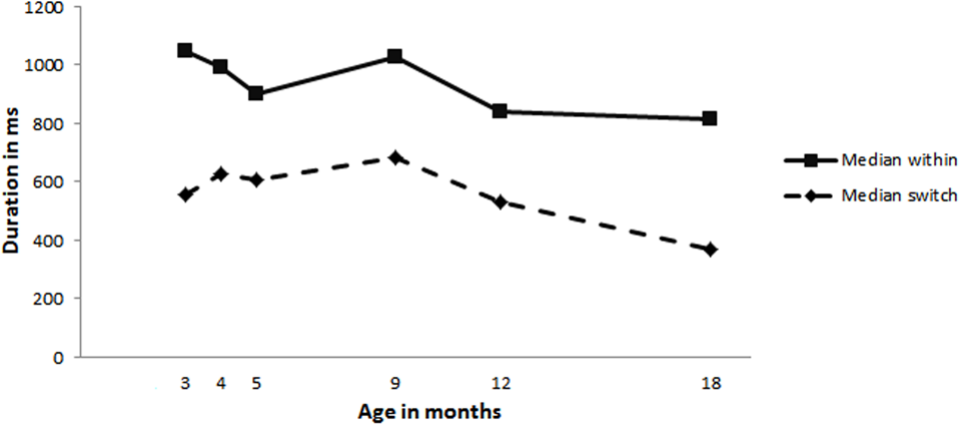
**Mothers’ median within-turn pause durations (solid line) and median gap durations after an infant vocalization (dashed line)**.

**FIGURE 7:**
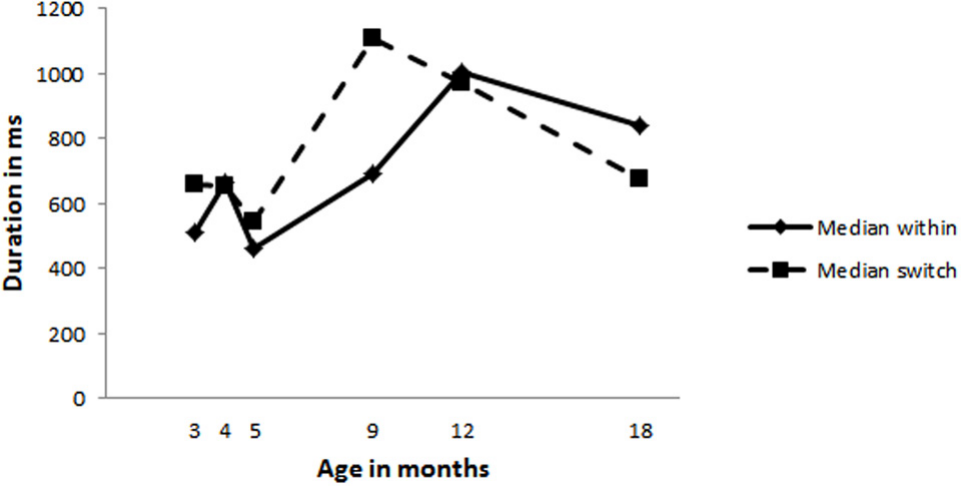
**Infants’ median within-turn pause durations (solid line) and median gap durations after an maternal vocalization (dashed line)**.

Next we analyzed whether mothers changed their within-turn pause durations, i.e., the silence between two consecutive utterances, to allow their infants more time to produce a turn. Maternal median within-turn pause durations did not change with increasing infant age (see **Figure 6** for median within-turn pauses and **Table 5** for the range in medians observed). However, as can be seen in **Figure 6**, mothers leave longer pauses after their own utterances (within-turn pause duration) compared to when they respond to an infant vocalization (switch), a pattern found in adult–adult interaction. Analyses conducted on the infant within-turn pause durations revealed a significant effect for age [χ^2^(1) = 27.65, *p* = 0.000; model: *loglikehood* = -15068, *N* = 1699]. The within-turn pause durations of the infants significantly increase with age, *OR* = 5.950459e+18, *p* = 0.000, indicating they wait longer after their own turn as they get older. Furthermore, as can be seen in **Figure 7**, infants do not yet leave longer gaps after their own vocalizations compared to after their mothers’ utterances.

**Table 5:** Range of median within-turn pause durations at each age in ms.

	Month 3	Month 4	Month 5	Month 9	Month 12	Month 18
Infant	307–1197.5	320–1208	360–1549	189–5760	420–1925	560–1590
Mother	799–1597	761–1325.5	659–1538	706.5–1559	565–1105	475–1650

## General Discussion

As reported above, we conducted a longitudinal study to explore the development of the timing of turn-taking in infancy. By using fine-grained temporal analyses of vocalizations, we were able to examine the structure and timing of vocal turns and their developmental trajectory in mother–infant exchanges during the first year and a half of life. Thus far, research on the timing of turn-taking, or turn-timing, in infancy has been fragmented, mostly cross-sectional, and has either looked at the development of overlapping vocalizations or gap durations but not both (e.g., Bateson, 1975; Jasnow and Feldstein, 1986; Elias and Broerse, 1996). Longitudinal designs including larger samples and tracking development over extended periods of time can provide valuable insights into stability and developmental change in turn-timing skills. We therefore analyzed mother–infant free-play interactions from 3 to 18 months. This allowed us to track the development of turn-timing from early in infancy to an age at which infants begin to acquire productive language. Our aim was to provide a more concise picture of the development of turn-timing, including both the development of overlapping vocalizations and gap durations. Based on previous findings (Garvey and Berninger, 1981; Casillas et al., under review) and the predictions made by the Interaction Engine Hypothesis (Levinson, 2006) we expected that the temporal aspect of turn-taking would develop early in infancy but that, when infants first start to produce language, their timing would slow down. In addition, we explored whether the turn-timing patterns in the mother–infant exchanges were reciprocally structured. As described, we transcribed 10-min free play interactions of 12 mother–infant dyads at the ages of 3, 4, 5, 9, 12, and 18 months, and extracted all turn transitions from these transcriptions. Next we analyzed the turn transitions for the developmental patterns of overlaps, gaps and the transitions as a whole (i.e., both gaps and overlaps).

### Overlap

Infants started out by producing just over a third of their turns in overlap with their mothers. This amount remained stable across 3, 4, and 5 months. However, between 5 and 18 months the amount of overlap decreased to about a fifth of their turns. Maternal overlap remained relatively stable over time and at 18 months the percentage of turns produced in overlap by infants was at a similar level to that of their mothers. This finding suggests that from 5 months onward infants adopt more of a turn-taking-like structure in interactions with their mothers. These findings are similar to previous studies reporting a decrease in overlapping vocalizations from 3 to 6 months and onward (Elias and Broerse, 1996) and around 4 months of age onward (Ginsburg and Kilbourne, 1988). In our dataset this decrease in overlapping vocalizations occurred slightly later, which could be due to differences in sample size. For example, Ginsburg and Kilbourne followed three dyads focusing on the period between 2.5 and 5 months. Instead of a decrease, Rutter and Durkin (1987) observed an increase in overlapping vocalizations from 9 to 24 months of age. However, they suggested that this might be due to the increasing amount of vocalizations that infants produced in general. In the present study we analyzed the amount of overlap by calculating the percentage of turns produced in overlap, therefore accounting for differences in total number of turns.

Even though the present data shows that infants start to decrease the amount of overlap that they produce, this does not mean that infants, like adults, aim for the end of the previous turn as the place to launch their own turn (Sacks et al., 1974; Jefferson, 2004). If that were the case we should also see that infants produce shorter overlap durations as they get older. We therefore analyzed the median overlap durations. These analyses revealed that infant overlap durations remained stable with age, while overlap durations of the mothers decreased with infant age. To summarize, mothers and infants start to adopt a more turn-taking like pattern in vocal exchanges from around 5 months onward as evidenced by the decrease in the percentage of overlap that infants produce. However, when infants do produce their turns in overlap they do not yet seem to be aiming, like adults, for the end of the turn.

### Gap

Analyses on the median gap durations revealed that the gap durations of the mothers remained fairly stable over time, but infants’ gap durations became significantly longer with age. Further exploratory analyses comparing infant gap durations at 5 months with the gap durations at 9, 12, and 18 months respectively revealed that infants were significantly faster at 5 months compared to at 9 and 12 months, but not at 18 months. Together, these findings suggest that infants are initially relatively fast in responding to their mothers’ turn, but slow down considerably at 9 months after which they start to pick up speed again. This is consistent with our expectations based on previous work and the Interaction Engine Hypothesis (Garvey and Berninger, 1981; Levinson, 2006; Casillas et al., under review). However, infants start slowing down somewhat earlier than expected. We expected that the slowing down would coincide with the emergence of productive language, which would be around 12 months instead of at 9 months. Our reasoning was that infants would slow down when they need to integrate these developing linguistic skills with their existing interactional timing skills and 9 months would be somewhat too early for this to occur. An alternative explanation for this finding might be found in infants’ changing communicative understanding. The period from 9 months onward is an age at which it has been shown that several skills relevant to communication are emerging, such as joint attention and pointing (e.g., Bakeman and Adamson, 1984; Carpenter et al., 1998). Furthermore, research has shown that this is the period in which infants begin to see others as intentional agents, which is suggested to be the prerequisite for word learning (Carpenter et al., 1998). In other words, infants at this age start to acquire an understanding of the communicative and shared function of social interactions. Thus, the finding that infants are slowing down in their turn-timing around 9 months of age might not be as surprising, as it occurs at an age at which infants are expected to be increasing their communicative understanding of social exchanges. However, more research is necessary to establish whether infants changing communicative understanding is indeed related to the slowing down observed at 9 months. This hypothesis is currently under further exploration. Another explanation might be found in a relation between the decrease in the amount of overlap and the increase in gap durations. It is possible that the decrease in overlap reflects some basic understanding of turn-taking, i.e., waiting to launch your turn until your interlocutor is finished speaking. This in turn could cause the increase in gap duration because infants are waiting until the end of their mother’s turn. However, when infants produce overlap at 9 months and older they do not show a decrease in the durations of these overlaps, which is what you would also expect if infants are waiting until the end of the turn. Nevertheless, the possibility that the increase in gap durations is related to the decrease in overlaps deserves further study.

### Reciprocity

Work by Anderson et al. (1977), Jasnow and Feldstein (1986), Beebe et al. (1988), Jaffe et al. (2001), showed that infants at 4 months adjusted their gap durations on the basis of who they interacted with. Furthermore, they showed that at 9 months maternal gap durations were influenced by infant gap durations and vice versa. Based on this earlier work we expected to find evidence of reciprocally structured turn-timing patterns at all ages. This is exactly what we found. In the present study maternal turn-timing remains fairly stable over time, while infant turn-timing is changing considerably: the amount of overlapping vocalizations decreases with infant age, while their gap durations seem to be much longer around 9 and 12 months of age compared to the gap durations at 3, 4, and 5 months. But, even though this indicates that infants are changing their turn-timing behavior over time, it is still possible that infants were vocalizing randomly while mothers were mainly responsible for establishing a turn-taking structure. Mothers could have changed, for example, how long they will wait for their infant to produce a turn after their own utterances, i.e., the duration of mothers’ within-turn pause. Longer maternal within-turn pause durations could explain a decrease in infant overlapping vocalizations because infants are given more time to respond. We therefore ran two analyses to assess the reciprocity of the vocal exchanges. We first analyzed whether the observed distribution of median durations of turn transitions was significantly different compared to randomly sampled distributions. This analysis revealed that the observed data was different from random distributions at all ages. Next we analyzed maternal within-turn pause durations. This revealed that mothers do not change their within-turn pause durations with increasing infant age. They wait equally long for an infant turn regardless of infant age. Thus the decrease in the percentage of turns that infants produce in overlap cannot be explained by a change in maternal within-turn pauses. Mothers do produce longer within-turn pauses compared to their gap durations when responding to an infant vocalization, which is similar to what Bateson (1975) found in her observation of mother–infant vocal exchanges. This suggests that in general, at all observed ages, mothers respond faster after an infant vocalization compared to after their own utterances. Bateson also observed a similar trend for the infant in her study, which is different from what we observed in the present data. This difference could be due to differences in definitions: Bateson calculated the within-turn pause from the onset of the utterance until the onset of the next utterance, i.e., including the preceding utterance, while in the present study within-turn pauses were calculated from the end of utterance until the beginning of the next utterance. In addition, there is also a difference in sample size: Bateson followed one dyad from 1.5 to 3.5 months while the present study followed 12 mother–infant dyads across a longer period of time. Our findings show clear changes in infant turn-timing skills which do not seem to be due to differences in maternal turn-timing as mothers remain stable with regards to the amount of overlap they produce, their gap durations and their within-turn pause durations. Infants thus seem to actively contribute to the observed changes in turn-timing.

The present findings provide some initial support for the Interaction Engine hypothesis, especially the findings with regards to the gap durations which are relatively short early in infancy but have increased considerably around 9 months. This slowing down coincides with a period of important changes in infants’ communicative and social understanding of interactions (e.g., Bakeman and Adamson, 1984; Carpenter et al., 1998). However, more research is needed to further explore what exactly is driving this change in turn-timing and whether this slowing down is related to infants’ advancing communicative understanding of interaction. Future studies should combine experimental methods that assess infants’ turn-timing skills with assessments (e.g., experiments or parental report) of infants’ language comprehension and production skills, to disentangle possible links between infant turn-timing and language production and between infant turn-timing and their understanding of language. Moreover, research should aim to assess whether the changes observed in timing in this study are related to infants’ changing understanding of the communicative and social function of social exchanges. One possible explanation for why infants are slowing down we are currently exploring, is whether the complexity of infant vocalizations might be related to turn-timing. A recent study by Casillas et al. (under review) has demonstrated such complexity effects on timing in older children. Furthermore, the present study solely focused on turn-timing in vocal exchanges, while turn-taking, and thus turn-timing, occurs from early on in infancy in various types of social exchanges, not necessarily just in vocal exchanges. For example, give and take sequences involving objects occur from around 9 months of age onward (e.g., Bates et al., 1975). Another option would be to look at the timing of pointing which emerges between 9 and 12 months. It could be, for example, that infants respond faster when pointing compared to when using a vocal response. Analyses of the timing involved in these types of turn-taking sequences might give additional insights into the role of timing in interaction in general versus timing specifically related to vocal exchanges.

The present study found no indication that changes in maternal timing were responsible for the changes in infant timing. However, mothers might have been changing other behaviors that influenced infant timing, including the use of gestures, facial expressions or changes in the content of the exchanges. For example, a mother could lean forward toward her child as to indicate ‘I am handing you the turn,’ which could facilitate turn-timing. But, if such cues were influencing the vocal-timing assessed in the present study one would expect that infants are becoming better with age at interpreting these cues and therefore will speed up with age instead of slowing down. Nevertheless, future studies should address the use and role of multimodal cues on turn-timing. The use of motion sensors could allow for conducting analyses at the same fine-grained level as with vocal turn-timing in the present paper. In addition, questions remain on how much of the changes in infant timing are due to social interactional experience. Based on studies assessing short-term effects of contingent and non-contingent interaction on infant behavior it seems likely that early interactional experience plays an important role (e.g., Bloom et al., 1987; Bloom, 1988; Masataka, 1993). Nevertheless, more research is needed to further explore the role of social interactional experience in the first few months of life on the development of infant turn-timing skills. For example, short training studies in which parents are trained to provide contingent feedback could provide insights into the impact of contingent experience in interaction on infant turn-timing skills (e.g., McGillion et al., 2014). In addition, studies including different types of samples, such as infants of mothers who suffer from postnatal depression, can also shed light on the role of interactional experience (e.g., Field, 1984). Finally, research should not ignore the infants’ possible role in the development of early interactional skills. Infants are likely to differ in how many opportunities they provide for their mothers to respond, by gazing, smiling, and vocalizing at their mother. Thus, individual differences in infant characteristics could also play a role in infants’ interactional experiences and the development of infant turn-timing.

The present study is, to our knowledge, the first study to assess turn-timing in infancy including both overlap and gap. In addition, we believe the present study is the first to provide a comprehensive overview of this development including not only analyses on the amount of overlapping vocalizations, but also assess the duration of overlap. The longitudinal design of the study has allowed us to demonstrate that infants’ turn-timing skills are changing considerably during infancy and that these changes occur around the same time as when infants’ communicative understanding has been found to be changing (e.g., Bakeman and Adamson, 1984; Carpenter et al., 1998). Furthermore, maternal turn-timing does not change much over this period of time indicating that the infants are actively involved in this observed developmental change. The observed developmental pattern is consistent with earlier research (e.g., Garvey and Berninger, 1981; Ginsburg and Kilbourne, 1988) and the predictions of the Interaction Engine Hypothesis (Levinson, 2006). Finally, the finding that infants are relatively fast turn-timers at 3, 4, and 5 months highlights the existence of remarkable social interactional abilities early in infancy.

## Conflict of Interest Statement

The authors declare that the research was conducted in the absence of any commercial or financial relationships that could be construed as a potential conflict of interest.
